# Effect of Silvopastoral Systems in the Thermoregulatory and Feeding Behaviors of Water Buffaloes Under Different Conditions of Heat Stress

**DOI:** 10.3389/fvets.2020.00393

**Published:** 2020-07-17

**Authors:** Maykel A. Galloso-Hernández, Vicente Rodríguez-Estévez, Carlos A. Alvarez-Díaz, Mildrey Soca-Pérez, Devon Dublin, Jesús Iglesias-Gómez, Leonel Simon Guelmes

**Affiliations:** ^1^Department of Animal Production, Universidad de Córdoba, Córdoba, Spain; ^2^Department of Basic Sciences, UACA Universidad Técnica de Machala, El Oro, Ecuador; ^3^Department of Sustainable Systems, EEPF: Indio Hatuey, Matanzas, Cuba; ^4^Global Education Leadership Program, Hokkaido University of Education, Kushiro, Japan

**Keywords:** buffaloes, silvopastoral system, feeding behavior, thermoregulatory behavior, *Leucaena*

## Abstract

Buffaloes use wallowing behavior to release excess heat in tropical conditions. The aim of this study was to evaluate the impact of silvopastoral systems in the feeding and thermoregulatory behavior of water buffaloes under moderate and intense heat stress. The behavior of water buffaloes was evaluated in two different production systems. The conventional system with Guinea grass (*Megathyrsus maximus*) only, and the silvopastoral system with Guinea grass and *Leucaena leucocephala* trees. The relation between the frequency of animal activities and the length of time the animals engaged in each activity was measured during the day time (6:00–18:00 h) by visual observations at 10-min intervals. The results obtained suggest that buffaloes use tree shade to partially supplement wallowing. Feeding behavior increased under intense heat stress in the silvopastoral system indicating that it can be a promising alternative to improve the buffaloes rearing conditions in the tropics.

## Introduction

The thermoregulatory behavior of water buffaloes in the tropics includes wallowing in water and shading (1, 2). The reduction in water reservoirs due to climate change can have a negative impact on buffalo production systems where wallowing is essential for thermoregulation (3). Heat stress reduces grazing and as a consequence, a reduction in the productive and reproductive indicators in water buffaloes (4). Previous studies in tropical environments revealed that silvopastoral systems influence the productivity of animals and their response to heat stress (5–7). The combination of leguminous trees and pastures increases 3-fold buffalo production per hectare when compared to systems that do not include trees (8). Trees increase the availability of food (pasture and tree leaves) (9), the nutritional value of grass, and improve soil characteristics by contributing to nitrogen fixation (10, 11). In addition, the inclusion of trees in grasslands has been linked to a decrease in parasitic diseases. For example, the inclusion of *Leucanea* trees reduces infection by *Haemonchus* and *Ostertagia* in buffaloes (12, 13) due to the presence of condensed tannins and polyphenols with antiparasitic effect in these trees (14).

It was suggested, that the use of leguminous trees in silvopastoral systems can replace the need for bathing water, thus becoming a promising alternative in tropical conditions where water is a limiting factor (8, 15, 16). A comparative study showed that a silvopastoral system based on *Leucaena* increased the weight of buffaloes in the early growing stage, a result that was not observed with bovines (17). Furthermore, Iglesias et al. (18) found better average daily weight gain rates for buffaloes in silvopastoralism, without including water for wallowing. This suggests that silvopastoral systems can reduce the need to provide water intended for wallowing to address heat stress.

The thermoregulatory physiology of buffaloes is limited due to the low number of sweat glands per square centimeter and the dark color of their skin that prevents them from thermoregulating efficiently (1, 19, 20). As an evolutionary adaptation, buffaloes express thermoregulatory behavior that includes wallowing in mud or water in flooded zones, or lying down under tree shade (21). Other observations include the movement toward higher quality pasture located near river banks (22) and adjustments of their grazing hours to night hours (23, 24). Research conducted to evaluate alternatives meant to improve thermoregulatory behavior of buffaloes under different conditions, shows that the provision of shade is important (2, 8, 15, 16).

Despite these recent advances, the influence of silvopastoral systems on the feeding and thermoregulatory behavior of buffaloes in tropical conditions under moderate and intense heat stress remains unknown. We hypothesized that the use of trees in silvopastoral systems can reduce wallowing as a thermoregulatory behavior and positively influence feeding behavior. To answer this hypothesis, in this study we examined the influence of the presence of trees on the thermoregulatory behavior of buffaloes under conditions of intense and moderate heat stress. The results show that the use of trees decreases the expression of wallowing as a thermoregulatory behavior and increases the time spent on food consumption.

## Materials and Methods

### Ethics Statement

The experiment recieved the approval of the Scientific Council and the Ethics Committee of the “Indio Hatuey” Grass and Forage Experimental Station, Matanzas, Cuba. This study did not involve any harm or cruelty to the animals.

### Study Site and Animals

The study was conducted from 2007 to 2009 in the municipality of Périco, Matanzas, Cuba located at 22° 48'7” of latitude north and 81° 1'of longitude west and 19.01 meters above sea level. The experimental phase was carried out on hydrated red ferralitic soil (25). This soil is moderately acidic [5.60 pH (KCI)], low in phosphorus content (2.43 mg/100 g), contains 0.18% total nitrogen and 3.20% organic matter. Among the exchangeable cations, calcium predominates (11.84 meq/100 g); while the cation exchange capacity (CEC) is slightly low (19.21 meq/100 g), therefore it is considered as having medium fertility. The climate of the region is tropical, seasonally humid, with an annual average temperature between 24.3 and 33.4°C, and a relative humidity of 80%. The annual rainfall was 1,331 mm, where 79.8% of it occurs between May and October (26). Nine female water buffaloes (*Bubalus bubalis*) with an average weight of 167.9 kg and 12 months of age were used for the study. These animals were heifers in 2007, impregnated in 2008 and were lactating in 2009. The animals remained in the pasture during the day and were taken to a paddock at night. In the pasture area, access to water was provided for wallowing. Natural shade by *Dichrostachys cinerea* (Marabú) was available in the wallowing areas. Drinking water and mineral salts were provided *ad-libitum*.

### Experimental Design

**Figure 1:**
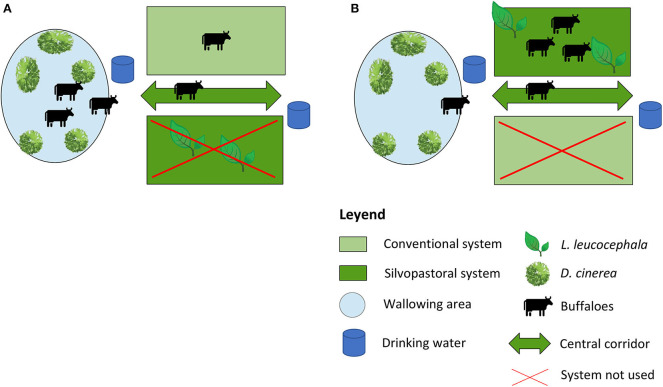
Management of the animals [treeless system **(A)** vs. silvopastoral systems **(B)**] in the study.

In this study, the influence of two production systems and different heat stress conditions on the thermoregulatory and feeding behavior of buffaloes were evaluated. The behavior of the animals in two productive systems was compared, the conventional one (i.e., only pastures without the presence of trees, Figure 1A) and the silvopastoral (i.e., pastures combined with trees, Figure 1B); under two conditions of heat stress, moderate (THI <75) and intense (THI> 75). The experiment followed the longitudinal analysis method in which thermoregulatory and dietary behaviors were observed in the same group of animals under four different experimental conditions (Table 1). Measurements were made in 12-h day cycles for three consecutive days in each experimental condition (T1, T2, T3, and T4). Before each measurement cycle, the botanical composition and pasture availability were analyzed. An adaptation period was included between each 36-h measurement cycle where no measurements were made on the animals for 24 days. At all times, the animals had access to water for wallowing. The total area was 7.33 ha divided into 12 paddocks of an average 0.54 hectare (ha, 10.000 m^2^) each. The layout of the experimental area is shown in Figure 1.

**Table 1:** Outline of experimental conditions.

**Treatments**	**Definition**	**Number of observations**	**Frequency (min)**	**Total time observed (hours)**	**Number of days**	**Number of observation cycles**
T1	Treeless system in intense thermal stress	1,872	10	312.0	29.0	9.6
T2	Treeless system in moderate heat stress	1,067	10	177.8	16.5	5.5
T3	Silvopastoral system in intense thermal stress	1,361	10	226.8	21.0	7.0
T4	Silvopastoral system in moderate thermal stress	1,226	10	204.3	19.0	6.0

### Description of Production Systems

The conventional system was based on grazing Guinea grass (*Megathyrsus maximus*). The silvopastoral system included Guinea grass and 600 *Leucaena leucocephala* trees per ha. The height of the *L. leucocephala* trees was between 1.7 and 2 m allowing the animals access to the branches as a food supplement. The animals had free access to drinking water and the wallowing area through a central corridor (Figure 1).

### Determination of Botanical Composition and Pasture Availability

The determination of the botanical composition was done using the step method described by Anon (27) and Sánchez et al. (28), which consisted of walking along the diagonals in each paddock, classifying for every three steps, the species of grass that coincided with the toe of the shoe. The grass with the highest predominance in the system (82–93% of vegetal cover) was *M. maximus*. Other less represented grasses were of the genera *Dichantrium* and *Brachiaria*. The botanical composition and grass availability were determined in order to calculate the grazing pressure [i.e., kg dry mater (DM)/100 kg body weight (BW)] and food offered (i.e., ton DM/ha). The DM of the grass was determined in the laboratory of the Indio Hatuey Grass and Forage Experimental Station. To estimate the DM content, 500 g of the homogenized grass samples was dried in a forced air oven at 60°C for 24 h. The weight was determined before and after the dehydration process (29) using an analytical balance of sensibility 0.001 kg.

The availability of the herbaceous stratum was determined by the visual estimation method using a 0.25 m frame according to the description by Martínez et al. (30). Eighty (80) measurements of grass height were taken in the grazing areas, at random, with a graduated ruler. After the average grass height was obtained, two frames were cut at approximately that height. The following equation was used to calculate availability: DC = (Dm x AA/AM) ^*^ 40 where DC: paddock availability, Dm: mean frame availability, AA: mean area height, AM: average height of the frames and factor 40 is the pasture availability within the frame. The initial values of grass availability were homogenized to 2.5 ton DM/ha by slash cutting the area 35 days before the animals entered the paddock.

The density of *L. leucocephala* was determined at the two periods of the year (rainy and dry). The plants encountered in three rows (each row measuring a distance of 30 m) were counted and an average per row was taken. The distance between each row was 10 m. The average number of plants in 30 m was multiplied by the distance between the rows giving rise to the average number of plants per 300 m^2^. This was used to determine the density of plants per ha.

### Temperature and Humidity Index

The temperature index (ITH) (31) was calculated using the formula (32): THI= (1.8 × T + 32) - [(0.55–0.0055 × RH) × (1.8 × T - 26)] where T is the air temperature (°C) and RH the relative humidity (%). Heat stress was classified as intense (THI> 75) and moderate (THI <75), as reported by Pérez et al. (33) for the conditions of Cuba. The conditions of intense heat stress in Cuba appear between the months of May to October and the conditions of moderate heat stress between the months of November to April. The environmental temperature and relative humidity of the site was measured for each treatment under the conditions of moderate heat stress and intense heat stress at the height of the withers in animals (1.7 m).

### Behavioral Observations

Measurements of feeding and thermoregulatory behaviors of buffaloes were performed using the direct observation method (34). The number of animals in each activity between each measurement interval was recorded. Each observation cycle consisted of 72 observations made over 3 days with a 10-min interval between observations from 6:00 to 18:00 h (Table 1). The animals were observed by an experienced observer, who maintained a secure distance which did not influence the behavior of the animals. Several activities were recorded, corresponding to grazing, browsing, rumination, water intake, shading (being positioned in the shade of trees) and wallowing (Table 2). The frequency of animals in each behavior per observation was recorded in an Excel database and the time spent on each activity was estimated. The time spent was calculated based on the application of Dumont and Petit (34) equation: Time spent in each activity = sum (ai x n)/A where **ai** is the number of animals that perform the activity, **n** the time between two successive observations and **A** the total number of animals. We then grouped the related variables. Active feeding behavior was considered as the sum of grazing and browsing. Feeding behavior was considered as the sum of active grazing behavior, rumination and water intake. Thermoregulatory behavior was considered as the sum of wallowing behavior and shading behavior (Table 2). For more comprensive, we show in Table 3, the fodder offered and grazing pressure in Silvopastoral system and conventional system.

**Table 2:** Description of recorded activities.

**Behavior activity**	**Definition**
**Active feeding behavior**	
Grazing	Time spent eating grass in the paddocks.
Browsing	Time spent in browsing understood as the process of consuming the tips of branches and tree leaves.
**Passive feeding behavior**	
Rumination	Time spent in rumination understood as the process of regurgitating previously ingested food and masticating it a second time.
**Thermoregulatory behavior**	
Wallowing	Time spent in wallowing understood as having a bath in the pond of water to cool.
Shading	Performing any activity under the trees (in the systems without trees it was possible in the wallowing area).
**Others**	
Water intake	Time spent in water intake understood consuming water in the central corridor.

**Table 3:** Fodder offered and grazing pressure in Silvopastoral system and conventional system.

**Treatment**	Offer of fodder (Dry Matter/ha) per rotation^*^	Grazing pressure^**^
T1	4.27 Ton/ha	8.52 kg
T2	2.19 Ton/ha	14.8 kg
T3	6.68 Ton/ha	23.07 kg
T4	3.92 Ton/ha	17.16 kg

* *Rotation*.

** *DM/100 kg body weight*.

### Statistical Analysis

The software SPSS® version 25 was used for statistical analysis (IBM Corp®). An analysis of variance (ANOVA) was applied to find the differences between the behaviors, taking into account the levels of intense and moderate heat stress and the type of system (silvopastoral and conventional). The analysis of variance was performed after checking the distribution of normality of the times dedicated to each activity with the Kolmogorov Smirnov test. The average time dedicated to each activity was compared by stress levels and systems with Duncan's multiple range comparison test, in order to detect the inequalities between the means.

## Results

### Conditions of Intense Heat Stress Increase Thermoregulatory Behavior and Decrease Feeding Behavior in Conventional Systems

The wallowing time in the conventional system under conditions of intense heat stress was significantly higher compared to the time of wallowing under conditions of moderate heat stress (*P* < 0.05). In the conventional system, the shading time under intense heat stress differed significantly from the conventional system under moderate heat stress conditions (*P* < 0.05). The grazing time under intense heat stress in the conventional system was significantly less compared to the conventional system under moderate heat stress conditions (*P* < 0.05). The rumination time in intense heat stress was significantly reduced compared to the conventional system under moderate heat stress (*P* < 0.05). Under conditions of intense and moderate heat stress in the conventional grazing system, the time of thermoregulatory behavior is longer under conditions of intense heat stress and differed under conditions of moderate stress. However, water intake, feeding behavior, and active feeding behavior did not differ significantly under conditions of intense and moderate heat stress in the conventional system (Table 4). The browsing behavior was not remarkable because in the treeless system they only had occasional contact with some branches of *D. cinerea*, so this behavior did not differ in the conventional grazing system.

**Table 4:** Thermoregulatory and feeding behavior in system without trees.

	**T1**		**T2**	
	**Mean (h)**	SD^*^	**Mean (h)**	SD^*^
Thermoregulatory behavior	3.94^c^	6.01	1.14^a^	3.36
Wallowing	1.69^c^	3.08	0.81ª	2.10
Shading	2.77^b^	3.77	1.61ª	3.22
Active feeding behavior^***^	*4.80^*a*^*	3.92	*5.06^*ab*^*	3.71
Feeding behavior	*7.78^*a*^*	2.84	7.59	2.13
Grazing	5.28^a^	3.63	6.12^c^	3.18
Browsing	0.08^a^	0.55	0.01^a^	0.26
Rumination	3.05^b^	3.55	3.70^c^	3.67
Water intake	0.33^b^	1.13	0.32^b^	1.08

a, b, c *Mean with different superscripts in same row differs significantly for P < 0.05*.

* *Standard deviation*.

*** *Active Feeding behavior*.

### The Silvopastoral System Does Not Affect the Global Thermoregulatory Behavior Under Intense Heat Stress

In this study, global thermoregulatory behavior was defined as the sum of time spent in shading and in wallowing and it was measured under conditions of moderate heat stress and intense heat stress. Under moderate heat stress conditions, the shading time did not show significant differences between the silvopastoral system and the conventional grazing system. However, under intense heat stress, differences were found in the time dedicated to shading. Specifically, under conditions of intense heat stress, the time spent dedicated to shading in the silvopastoral system was higher than the conventional grazing system (ANOVA, *P* < 0.05) (Table 5).

**Table 5:** Time spent in thermoregulatory behavior, wallowing and shading.

**Treatment**	**Thermoregulatory behavior**			**Wallowing**			**Shading**		
	**Mean (h)**	SD^*^	EE^**^	**Mean (h)**	SD^*^	EE^**^	**Mean (h)**	SD^*^	EE^**^
T1	3.94^c^	6.01	0.13	1.69^c^	3.08	0.07	2.77^b^	3.77	0.09
T2	1.14^a^	3.36	0.10	0.81^a^	2.10	0.09	1.61^a^	3.22	0.14
T3	3.78^c^	5.66	0.15	1.39^b^	2.92	0.08	3.17^c^	3.83	0.11
T4	1.83^b^	4.02	0.11	0.71^a^	1.96	0.06	1.90^a^	3.34	0.11

a, b, c *Mean with different superscripts in same column differs significantly for P < 0.05*.

* *Standard deviation*.

** *Standard error*.

Regarding wallowing, under moderate heat stress conditions, no significant differences were found between the silvopastoral system and the conventional grazing system (Table 5). However, under conditions of intense heat stress, the wallowing time in the conventional grazing system was significantly longer than in the silvopastoral system (ANOVA, *P* < 0.05).

Overall, the time spent on thermoregulatory behavior under moderate heat stress was greater in the silvopastoral system compared to the conventional system (ANOVA, *P* < 0.05). In contrast, the thermoregulatory behavior of animals in these grazing systems did not differ significantly under conditions of intense heat stress (Table 5).

### The Silvopastoral System Increases Active Feeding Behavior Under Intense Heat Stress

The silvopastoral system influenced the time spent on grazing under the two conditions of heat stress. Under moderate heat stress conditions, the time the animals spent grazing was less than in the silvopastoral system compared to the conventional system (ANOVA, *P* < 0.05). In contrast, under conditions of intense heat stress, the grazing time was longer in the silvopastoral system than in the conventional system (*P* < 0.05) (Table 6).

**Table 6:** Time spent in active feeding behaviors.

**Treatment**	Active feeding behavior^*^			**Grazing**			**Browsing**		
	**Mean (h)**	SD^**^	EE^***^	**Mean (h)**	SD^**^	EE^***^	**Mean (h)**	SD^**^	EE^***^
T1	4.80^a^	3.92	0.09	5.28^a^	3.69	0.09	0.08^a^	0.55	0.01
T2	5.06^ab^	3.71	0.11	6.12^c^	3.18	0.10	0.01^a^	0.26	0.01
T3	5.64^c^	4.05	0.1	5.60^b^	3.63	0.10	0.39^b^	1.04	0.03
T4	5.12^a^	3.94	0.11	5.54^ab^	3.48	0.10	0.47^b^	1.37	0.04

a, b, c *Mean with different superscripts in same column differs significantly for P < 0.05*.

* *Active feeding behavior is the sum of grazing and browsing*.

** *Standard deviation*.

*** *Standard error*.

The inclusion of *Leucaena* trees in the silvopastoral system increased browsing activity under moderate heat stress and intense heat stress compared to the conventional system where time dedicated to this activity was marginal (*P* < 0.05) (Table 6).

In this study, active feeding behavior was considered as the sum of the time spent on grazing and browsing. Under intense heat stress, the animals in the silvopastoral system had significant differences in the active feeding behavior when compared with the conventional system (*P* < 0.05). However, under moderate heat stress conditions, no differences were found between the silvopastoral system and the conventional system (Table 6).

Under moderate heat stress conditions, water intake in the silvopastoral system differed from the conventional system (*P* < 0.05). However, under conditions of intense heat stress, no differences were found between the silvopastoral system and the conventional system (Table 7).

**Table 7:** Time spent in different feeding behavior.

**Treatment**	Feeding behavior^***^			**Rumination**			**Water intake**		
	**Mean (h)**	SD^*^	EE^**^	**Mean (h)**	SD^*^	EE^**^	**Mean (h)**	SD^*^	EE^**^
T1	7.78^a^	2.84	0.06	3.05^b^	3.55	0.08	0.33^b^	1.13	0.02
T2	7.59^a^	2.13	0.06	3.70^c^	3.67	0.14	0.32^b^	1.08	0.04
T3	8.22^c^	2.33	0.06	2.58^a^	3.58	0.10	0.38^b^	1.18	0.03
T4	7.84^b^	2.25	0.06	3.31^b^	3.74	0.12	0.20^a^	0.87	0.03

a, b, c *Mean with different superscripts in same column differs significantly for P < 0.05*.

* *Standard deviation*.

** *Standard error*.

*** *Feeding behavior is the sum of grazing behavior, rumination and water intake*.

Feeding behavior generally showed significant differences under intense heat stress between the silvopastoral system and the conventional treatment. The values found in the active feeding behavior under moderate heat stress conditions also differed significantly between the silvopastoral system and the conventional system (*P* < 0.05) (Table 7).

### The Silvopastoral System Reduces Rumination Time Under Intense and Moderate Heat Stress

The time spent on rumination in the silvopastoral system under conditions of intense heat stress was significantly different from the conventional system (*P* < 0.05). Significant differences were found for rumination behavior under moderate heat stress between the conventional system and the silvopastoral system (*P* < 0.05) (Table 7).

## Discussion

Animal behavior studies with water buffaloes in production conditions are scarce and especially important in the tropics. Buffaloes have limited thermoregulatory capacity due to their low number of sweat glands and dark skin which makes them especially sensitive to heat stress (1, 35). In this study, we compared the thermoregulatory and feeding behavior of water buffaloes subjected to intense and moderate heat stress under silvopastoral and conventional systems. In particular, we were interested in evaluating how silvopastoralism influences the behavior of buffaloes under intense heat stress. Describing behavior in the same group of animals in a longitudinal study has as its main strength, the reduction of the individual effect as well as reducing the antagonistic behaviors that occur in the regrouping of the experimental groups in behavior studies (36, 37). However, one limitation of our work is the low number of animals used (*n* = 9).

The greater time spent on wallowing and shading in the conventional system under conditions of intense heat stress suggests an increase in thermoregulation needs (20). It was previously described in Brazil, through infrared thermography measurements, that extreme weather factors (THI> 80) affect buffaloes in the form of heat stress (35, 38). Other studies in India (1) and Thailand (21) also reflect these thermoregulation needs of buffaloes. It is possible that an increase in the thermoregulation needs reflected in the different wallowing and shading times, was due to the seasonality of the reproductive and productive performance of this species (4, 39, 40), as a consequence of the effect of the climatic seasonality present in Cuba and by extension the tropics (8). The decrease in thermoregulatory behavior was associated with a decrease in feeding behavior, which could be related to the heat stress to which the animals were subjected. Previous studies have shown that in intense heat stress, the times dedicated to grazing behavior and rumination decrease as a result of this discomfort in cattle (41).

In the conventional system, intense heat stress resulted in shorter grazing times despite the greater availability of pastures (6, 7, 42). This is relevant because it suggests that under intense heat stress conditions in buffaloes as well as cattle, feeding needs are just as important as heat defense needs (37). This shorter grazing time could also be related to the negative productive results reported in buffaloes in the tropics.

Remarkably, our study revealed that the silvopastoral system decreased the time dedicated to wallowing behavior under intense heat stress. This suggests that the silvopastoral system decreases heat stress in animals and improves their welfare. Previous studies in Cuba attributed these benefits to the microclimate generated under silvopastoralism by mitigating heat stress (5, 6, 18, 43). This was confirmed in our study when it was observed that the decrease in wallowing was associated with a significant increase in the time spent shading.

These results of high active feeding behavior and less passive feeding behavior in the silvopastoral system in heat stress conditions could be attributed to the presence of natural shade (10, 44). *L. leucocephala* and other tree species offer filterable radiation (natural shade). Additionally, the silvopastoral system contributes to a 10–30% increase of dry matter production in the form of grass (5). The different results reported in the productive yield per ha under silvopastoralism and the improvement of animal welfare could be related to those we found in our study. These are, the increase in feeding and thermoregulatory behavior under silvopastoralism and its influence on animal welfare (8, 15, 16, 18, 31, 41, 45, 46); and the decrease in rumination that we observed in the silvopastoral system attributed to the increase in nitrogen supply of the trees that increases ruminal microbial activity (11, 47), which was reported to be related to an improvement in ruminal function (48, 49).

It is possible that under situations of intense heat stress in silvopastoral conditions, the sensation of heat stress is not being perceived by the buffaloes, due to the contribution of trees in providing shade and improving active feeding behavior (50, 51). In the same way, shade in the silvopastoral system improved pasture quality and quantity in several studies (5, 10, 52).

In the silvopastoral system, under conditions of moderate thermal stress, the time spent on water consumption was reduced. This could be influenced by the composition of the grass (DM) that generate a level of satiety in the animals. Similar results reflected a 23% reduction in visits to the drinking source in cattle provided with shade (41). The similarities in water ingestion under conditions of intense heat stress suggest that the needs for water are more pressing than those for food, coinciding with Dukes (53) and Alvarez et al. (37).

## Conclusions

The silvopastoral system improved the thermoregulatory and feeding behavior under conditions of intense heat stress. This was reflected in the times spent shading and the increased grazing time in comparison with the conventional system where the wallowing time was greater. In the conventional system, the increase in grazing, rumination and wallowing under conditions of moderate heat stress, is a reflection of a greater difficulty to satisfy the thermal and food needs of water buffaloes. In the future, the effect that it would have on the thermoregulatory and feeding behavior of buffaloes without wallowing zones should be evaluated, under silvopastoral conditions.

## Data Availability Statement

The raw data supporting the conclusions of this article will be made available by the authors, without undue reservation.

## Ethics Statement

The animal study was reviewed and approved by the Ethics Committee of Experimental Station: Indio Hatuey, University of Matanzas, Cuba. Written informed consent was obtained from the owners for the participation of their animals in this study.

## Author Contributions

MG-H and LS: conceptualization and methodology. MG-H, MS-P, and JI-G: validation. CA-D, VR-E, and MG-H: formal analysis. MG-H, JI-G, MS-P, and LS: investigation. LS, MG-H, and MS-P: resources. MG-H, VR-E, MS-P, and DD: data curation and writing original draft preparation. DD, VR-E, and MG-H: writing—review and editing. MG-H and VR-E: visualization. VR-E and CA-D: supervision. MG-H and LS: project administration. All authors: read and agreed to the published version of the manuscript.

## Conflict of Interest

The authors declare that the research was conducted in the absence of any commercial or financial relationships that could be construed as a potential conflict of interest.
